# Idiopathic Hypertrophic Pachymeningitis: Does Earlier Treatment Improve Outcome?

**DOI:** 10.3390/children8010011

**Published:** 2020-12-28

**Authors:** Emilia Rizzo, Ailsa Elizabeth Ritchie, Vinay Shivamurthy, Ata Siddiqui, Ming Lim

**Affiliations:** 1 Faculty of Medicine and Surgery, University of Catania, Piazza dell’Università 2, 95124 Catania, Italy; rizzoemilia23@gmail.com; 2 Great Ormond Street Hospital for Children NHS Foundation Trust, London WC1N 3JH, UK; 3 Paediatric Ophthalmology, Evelina London Children’s Hospital at Guy’s and St Thomas’ NHS Foundation Trust, London SE1 7EH, UK; AilsaElizabeth.Ritchie@gstt.nhs.uk; 4 Paediatric Rheumatology, Evelina London Children’s Hospital at Guy’s and St Thomas’ NHS Foundation Trust, London SE1 7EH, UK; Vinay.Shivamurthy@gstt.nhs.uk; 5 Department of Neuroradiology, King’s College Hospital NHS Foundation Trust, London SE5 9RS, UK; ata.siddiqui@gstt.nhs.uk; 6 Children’s Neurosciences, Evelina London Children’s Hospital at Guy’s and St Thomas’ NHS Foundation Trust, King’s Health Partners Academic Health Science Centre, London SE1 7RS, UK; 7 Faculty of Life Sciences and Medicine, Kings College London, London SE1 7RS, UK

**Keywords:** dural thickening, cranial neuropathy, immunotherapy, sarcoid, steroids, visual failure

## Abstract

Background/goal: Hypertrophic pachymeningitis is a rare chronic inflammatory disorder characterized by marked fibrous thickening of the cerebral and/or spinal dura mater. This condition has largely been reported in adults, but there are very few reports in children. Methods: We describe a 14-year-old boy with idiopathic hypertrophic pachymeningitis, who presented with deteriorating vision on a background of severe headache. We evaluated pediatric cases of hypertrophic pachymeningitis and compared treatments and their relation to outcomes. Results: There are only eleven pediatric cases of hypertrophic pachymeningitis reported in the literature. In the patients treated with steroids either at presentation or subsequent relapses, a good response was reported. In the cases with delayed initiation of steroid treatment, this was often related to an incomplete recovery. In our patient, this delay may have contributed to his poor visual outcome. Conclusions: Early initiation of steroid treatment in children with idiopathic hypertrophic pachymeningitis may improve outcomes.

## 1. Introduction

Hypertrophic pachymeningitis is a rare inflammatory disorder characterized by localized or diffuse thickening of the cranial or spinal dura mater, resulting in progressive neurological deficits. The etiology of the pachymeningitis is thought to be immune in nature or secondary to other possible causes of dural reaction, such as infectious conditions (neurosyphilis, tuberculosis, fungal infections, syphilis, Lyme’s disease); collagen vascular disorders (granulomatosis with polyangiitis, rheumatoid arthritis, systemic lupus erythematosus, mixed connective tissue disease); neoplasia (dural carcinomatosis, meningioma en plaque, lymphoma) and miscellaneous disorders such as sarcoidosis, mucopolysaccharidosis, intracranial hypotension syndrome and intrathecal drug administration [1,2]. IgG4-related hypertrophic pachymeningitis (IgG4-RHP), a recently described entity, is an increasingly recognized manifestation of IgG4-related disease, a fibroinflammatory condition that can affect virtually any organ [3]. The present report describes a 14-year-old boy with idiopatic hypertrophic pachymeningitis, the course of his illness, his response to treatment and a comparison to the other cases reported in literature.

## 2. Case Study

A previously healthy 14-year-old boy presented with a several-week history of deteriorating vision on a background of experiencing headaches 9 months prior, which worsened over recent months. He reported double vision and progressive loss of vision in the right eye over the few weeks prior to presentation. There were no other constitutional symptoms like fever, night sweats or weight loss. The patient reported a non-specific insect bite 3 months prior, and there was no history of recent foreign travel.

The key finding on clinical examination was a fulminant right optic neuritis, where he could only perceive light, alongside right sided 4th and 6th cranial nerve palsy. There was no evidence of uveal disease. He had a full neurological examination, which did not reveal any abnormality. His systemic examination was normal and in particular there was no evidence of lymphadenopathy, organomegaly or cutaneous features of connective tissue disease.

**Figure 1:**
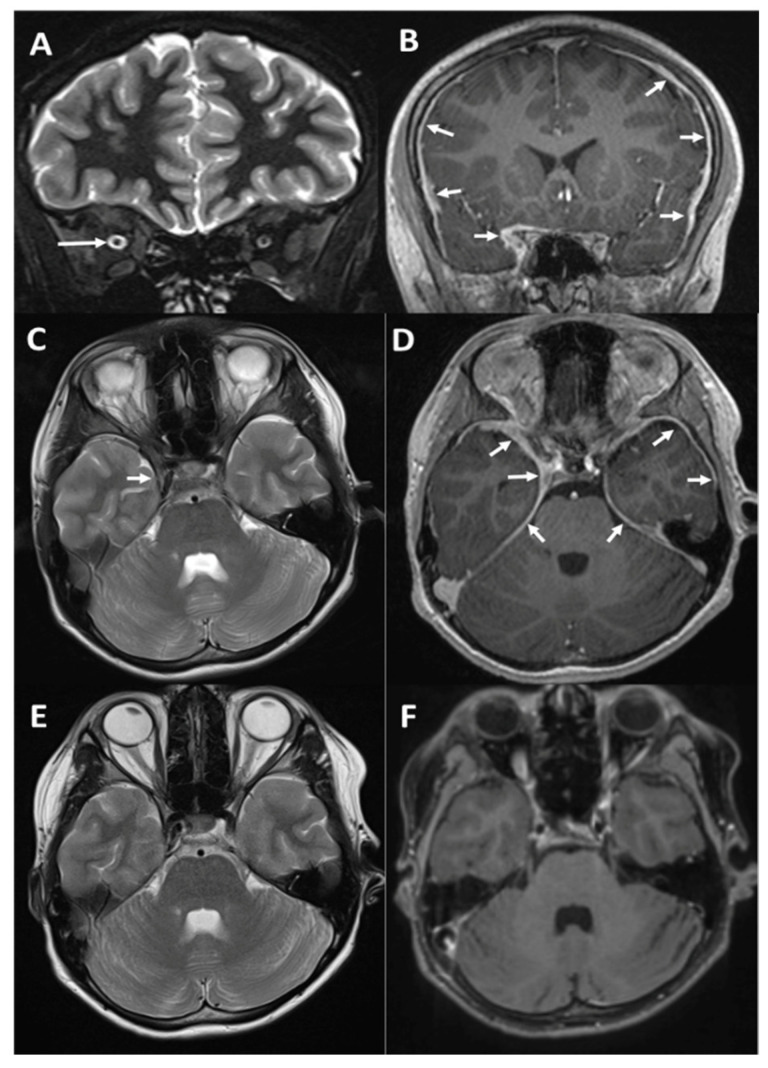
MRI findings in our patient. **Top row** (**A**,**B**): Coronal STIR image at presentation showing mild congestion of the right orbit with a dilated optic nerve sheath but normal signal of the optic nerve ((**A**), long arrow); coronal post-contrast T1w (T1 weighted) image showing diffuse dural thickening extending to the right cavernous sinus and optic canal, where it surrounds the optic nerve ((**B**), small arrows). **Middle row** (**C**,**D**): Axial T2w (**C**) and post-contrast T1w (**D**) images showing normal brain appearances but diffuse bilateral dural thickening with subtle corresponding low signal on T2. **Bottom row** ((**E**,**F**)): Follow-up imaging after a month showing resolution of the dural enhancement, though there is still some evidence of right 6th cranial nerve palsy (note the adducted right globe).

Magnetic resonance imaging (MRI) identified widespread dural (pachymeningeal) thickening with contiguous involvement of the cavernous sinus on the right side (Figure 1) with no evidence of brain parenchymal or white matter involvement.

Following consultation with the infectious diseases and rheumatology team, investigations were initiated which did not reveal an infective or immune etiology for the presumed pachymeningitis (Table 1).

High-dose pulsed intravenous methylprednisolone 30 mg/kg/day was administered for 3 consecutive days and his vision began to improve (counting fingers), along with a vast improvement in his headaches. He was converted to 60 mg prednisolone orally daily. Although dural biopsy was considered, a marked improvement in his imaging (Figure 1) to a near resolution of the lesion did not present us with a biopsy target. Similarly, systemic imaging with a whole-body MRI also failed to identify a potential lesion to biopsy or any underlying systemic process.

**Figure 2:**
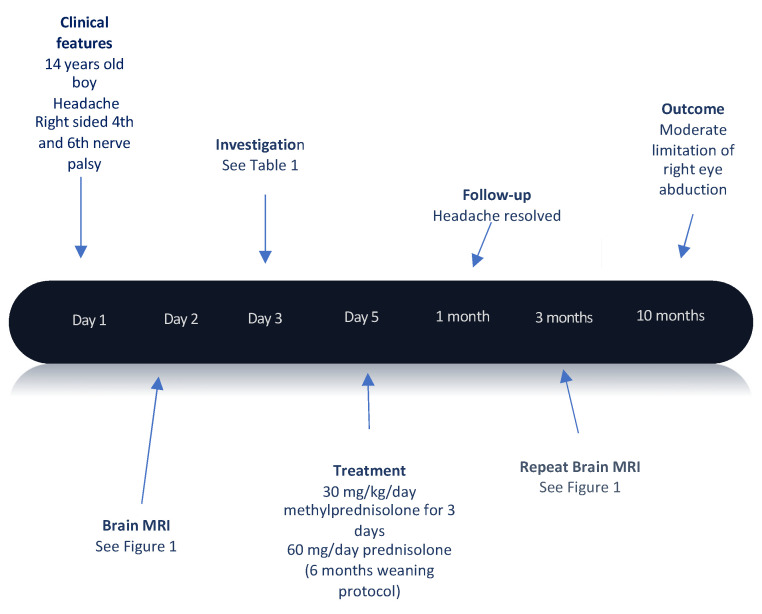
Timeline of our patient’s history.

He was reviewed in the outpatient clinic a month after discharge and was doing well. His headaches had resolved completely (timeline detailed in Figure 2). Ophthalmology assessment revealed an ongoing reduction of vision in the right eye with a central visual acuity of LogMAR 1.52 (2/60) and remaining limitation of abduction in the right eye to the mid-line. His optical coherence tomography showed retinal nerve fiber layer thinning in the right eye and a stable, normal retinal nerve fiber layer in the left eye.

At 3 months from symptom presentation, while on a slow steroid taper (10 mg daily, 6 months weaning protocol), repeat imaging continues to demonstrate no active dural disease. At latest follow-up (10 months), he remains well clinically and his visual acuity has improved to LogMAR 0.50 (6/19) in the right eye. His eye movements also continue to improve, with only 25% limitation of right abduction and he has no esotropia or double vision when looking straight ahead. He continues to have a mild right relative afferent pupillary defect and a pale right optic disc.

## 3. Discussion

Hypertrophic pachymeningitis was first described by Charcot and Joffroy in 1869 [4]. It is a rare chronic inflammatory disorder characterized by marked thickening of the cerebral and/or spinal dura matter. It has been described in association with infection, trauma, tumors and immune-mediated disease such as granulomatosis with polyangiitis, neurosarcoidosis or IgG4 related disease [1,2,3,5]. In cases when no causative factors can be identified, the condition is referred to as ‘idiopathic” [6].

The thickened dura causes progressive neurological impairment. Chronic headache and multiple cranial neuropathies are the most common clinical manifestations [5,6]. These may include ataxia, facial pain, cranial nerve involvement and neuro-ophthalmic complications such as papilledema and various neurological deficits [1,2].

The diagnostic workup should include serology tests for VZV, *Mycoplasma pneumoniae*, *Haemophilus influenzae type B*, HTLV1(Human T-cell lymphotropic virus type 1), HHV6(Human Herpes Virus 6), *Enterovirus*, *Borrelia burgdorferi*, CMV(cytomegalovirus), HIV(Human Immunodeficiency Virus), syphilis, EBV(Epstein-Barr Virus) and, in endemic areas, tuberculosis tests (Quantiferon tests and/or Mantoux skin testing). Moreover, an autoimmune screening is recommended, including neuronal surface antibody research and a complete CSF (Cerebral Spinal Fluid) exam.

The definitive diagnosis is based on MRI or brain biopsy of the thickened dura mater, which reveals interstitial fibrosis and inflammatory cell infiltration.

This condition has largely been reported in adults, but there are very few reports in children. We performed a review of the literature (Medline 1990–present) using the search terms (hypertrophic pachymeningitis) AND (paediatric OR pediatric OR children OR childhood). Twelve cases referring to children (<18 years old) were identified and the full manuscripts were available for review [7,8,9,10,11,12,13,14,15,16,17,18].

Among the twelve pediatric cases of hypertrophic pachymeningitis reported in the literature, three cases developed in association with tuberculosis [10,11,17], one was PR3-ANCA-associated [13], two developed in association with IgG4-RD [16,18] and six were regarded as idiopathic [7,8,9,12,14,15]. The clinical features, MRI findings, biopsy results, treatment and outcomes are summarized in Table 2.

In the patients treated with steroids either at presentation or subsequent relapses, a good response to steroids was reported (6 events; Case 2 relapse (R) 1; Case 3 presentation (P) and R1; Case 5 P; Case 7 R1; Case 9 P; Case 12 P and R1).

However, the course of the disease was chronic and relapsing in six of the 13 cases, necessitating more aggressive immune therapy such as methotrexate, cyclophosphamide, intraventricular cytarabine, azathriopine or rituximab (Cases 2, 3, 6, 7, 9 and 12).

Of the two patients (Cases 1 and 2) treated conservatively, one patient relapsed, who then responded to steroids (Case 2). Overall, all but one case (Case 6), had a satisfactory outcome (mRS 0–2). Residual visual deficits occurred in two cases and hypoglossal palsy in one case (Cases 3, 5 and 9); one chronically deteriorated neurologically into a chronic vegetative state (Case 6).

Although immunotherapy-responsive, the delay in initiating steroid treatment in our patient may have contributed to his poor visual outcome. In the reported cases with a lag in initiating steroid treatment, this was often related to an incomplete recovery (Case 2 initiating 21 months later at relapse; Case 3 6 months from prior episode managed as viral meningitis; Case 6 10 weeks).

In conclusion, idiopathic hypertrophic pachymeningitis can present in children and should be considered in cases of pachymeningitis and negative findings from diagnostic evaluation.

Early initiation of steroid treatment may improve the outcome.

## Figures and Tables

**Table 1:** Investigations excluding infectious and immune etiology of pachymeningitis in our case.

Infective Etiology	Serology/PCR *	
	VZV, *Mycoplasma pneumoniae*, *Haemophilus influenzae* type B, HTLV1 *, HHV6 *, *Enterovirus* *, *Borrelia burgdorferi*, CMV *, HIV, Syphilis, EBV	Negative
Immune-Mediated Disease	Autoimmune screening	
	ANA, Anti-dsDNA, Anti-SSA, Anti-SSB, Anti-phospholipid, Anti-cardiolipin, ANCA	Negative
	Neuronal surface and glial antibodies	
	MOG, Aquaporin 4, Glycine receptor	Negative
	CSF	
	WBC/mm^3^	<1
	Protein (mg/dL) and Glucose (mg/dL)	Normal
	Serum ACE level	44 (normal)
Other Investigations	Chest XR	Normal
	Abdomen US	Normal

HTLV1: Human T-cell lymphotropic virus type 1; HHV6: Human Herpes Virus 6; CMV: cytomegalovirus; HIV: Human Immunodeficiency Virus; EBV: Epstein-Barr Virus; MOG: Myelin-oligodentrocyte glycoprotein; CSF: Cerebral Spinal Fluid; asterisk by PCR would indicate that those with asterisk indicate PCR tersting.

**Table 2:** Clinical data of 13 patients with hypertrophic pachymeningitis.

Case Report	Clinical Features and Course	MRI	Investigation and Aetiology	Treatment	Outcome (mRS Score)
Case 1 Asano et al. (1998) 13 years F [7]	P: Headache, vomiting and aplastic anemia	P: Enhancement T1: Thickening of dura mater in tentorial region	Brain biopsy ND Idiopathic	P: Conservative	Full recovery, 6 months (mRS 0)
Case 2 Van Toorn et al. (2008) 10 years M [8]	P: Right 6th nerve palsy R1 21 months: Progressive cranial polyneuropathies	P: Normal R1: Enhancement T1: Widespread dural thickening	Brain biopsy ND Idiopathic	P: Conservative R1: IVMP; prednisone; MTX	Residual small angle esotropia, 1 year (mRS 1)
Case 3 Aburahma et al. (2009) 3 years 6 months M [9]	P: Headache, vomiting and irritability. R1 1 month: Progression of pachymeningitis R2 3 months: Headache, vomiting and irritability. Decline visual activity R3 6 months: Headache, ataxia, poor vision and focal seizures.	P: Enhancement T1 in tentorium cerebelli and spinal meninges; non-communicating hydrocephalus R1–R3: increasing meningeal enhancement	Brain biopsy: thickened and fibrotic dura mater Idiopathic	P: IVMP, prednisone R1: Steroids R2: CP R3: Intratecal cytarabine, hydrocortisone	Disinhibition, hyperactivity, poor vision and mildly ataxic gait, 3 years (mRS 2)
Case 4 Karimi et al. (2013) 4 years M [10]	P: Fever, meningism and left hemiparesis	P: Meningeal enhancement in frontal lobe; T10–T11 mass	Brain biopsy ND Spinal biopsy: tuberculous	P: TB therapy	Full recovery, 4 weeks (mRS 0)
Case 5 Sharma et al. (2014) 2 years 11 months F [11]	P: Fever, bilateral visual loss, left 3rd and 7th palsy	P: Enhancement T1 dura mater; cavernous sinus thrombosis; multifocal acute infarcts	Brain biopsy ND QuantiFERON-TB +ve	P: TB therapy; steroids	Residual mild left ptosis, 6 months (mRS 1)
Case 6 Tsuchida et al. (2018) 3 years F [12]	P: Headache, vomiting and weakness Weight loss; R1 10w: Seizure and encephalopathy R2 4 months: Lost of light reflex and spontaneous respiration	P: Gadolinium enhancement T1: widespread thickening of dura; communicating hydrocephalus R1–R2: Progressive dural thickening and brainstem lesions	Brain biopsy: thickened and fibrotic dura mater Idiopathic	P: Conservative R1–R2: IVMP, CP intrathecal cytarabine	Chronic vegetative state prior to death, 4 months (mRS 6)
Case 7 Matsumoto et al. (2018) [13] 14 years F	P: fullness and pain in her right ear R1: headache and dysarthria	P: mastoiditis involving the right temporal bone R1: extensive hypertrophy of the right hemispheric dura mater	Brain Biopsy: prominent inflammation and severe fibrosis. PR3-ANCA positive	P: Mastoidectomy + steroids + MTXR1: Steroids + CP	Full recovery, 1 month (mRS 0)
Case 8 Brand et al. (2018) 13 years M [14]	P: Headache, left 6th palsy R1 3w: Right eye diplopia	P: nodular dural enhancement and thickening in the right cerebrum, right cavernous sinus, and basilar cistern.	Brain biopsy: atypical lymphocytic infiltrate Idiopathic	P: IV cancomycin, ceftriaxone, and azithromycin R1: Conservative	Full recovery, 1 month (mRS 0)
Case 9 Hsieh et al. (2019) 16 years F [15]	P: Headache, right 12th nerve palsy R1 2w: Headache, right 12th nerve palsy	P: Gadolinium enhancement T1: Posterior fossa pachymeningeal thickening R1: No improvement of dural thickening	Brain biopsy: dural mixed inflammation Idiopathic	P: Steroids R1: Rituximab	Residual hypoglossal palsy, 2 years (mRS 1)
Case 10 Nambirajan et al. (2019) 16 years M [16]	P: right-sided focal seizures	P: large extra-axial contrast-enhancing lesion in the left frontoparietal region	Brain biopsy: inflammatory infiltrate along the dura–brain interface and predominated in histiocytes; IgG4-positive plasma cells IgG4-RD	P: Mass excision	Full recovery, 4 months (mRS 0)
Case 11 Sharawat et al.(2019) [17] 4 years F	P: left hemiparesis, communicating hydrocephalous R1: Left-sided ptosis, incomplete ophtalmoparesis, left-sided facial paresis, left hemiparesis	R1: mid-brain lesions, left-sided subdural collection, diffuse hypertrophic pachymeningitis, encephalomalacic changes in the right basal ganglia	Brain biopsy ND Tuberculous	P: TB therapy R1: TB therapy, steroids	Data not available
Case 12 Vakrakou et al. (2020) 17 years F [18]	P: mild upper limb weakness and a loss of dexterity with left hand R1 2 years: numbness of lower limb	P: Spine-longitudinal intramedullary damage, extending to the body of the C5 vertebra; Brain high-intensity lesions in the deep white matter, enlargement of hypophysis with homogeneous gadolinium enhancement R1: decline in lesion size and pituitary gland signal intensity	Brain biopsy ND IgG4-RD	P: Steroids + azathioprine R1: Steroids	Full recovery, 5 years (mRS 0)
Case 13 Our case 14 years M	P: Headache, double vision and visual loss; right 2nd, 4th and 6th nerve palsy	P Gadolinium enhancement T1: Thickening of dura mater and cavernous sinus on the right	Brain biopsy ND	P: IVMP, prednisolone	Moderate visual loss right eye, no esotropia, 10 months (mRS 1)

M is Male and F female; CP: cyclophosphamide; IVMP: intravenous methylprednisolone; MTX: methotrexate; ND: not done; P: presentation; R: relapse; TB: tuberculous; IgG4-RD: IgG4-related cisease.

## Data Availability

Authors will be happy to share anonymized clinical data additional to those provided in manuscript uopn request.
